# The Model Study of Phase-Transitional Magnetic-Driven Micromotors for Sealing Gastric Perforation via Mg-Based Micropower Traction

**DOI:** 10.3390/nano14100865

**Published:** 2024-05-16

**Authors:** Kang Xiong, Leilei Xu

**Affiliations:** State Key Laboratory of Advanced Technology for Materials Synthesis and Processing, Wuhan University of Technology, Wuhan 430070, China; kangxiong@whut.edu.cn

**Keywords:** micromotor, gastric perforations, magnetically driven, phase transition, magnetothermal, biocompatible

## Abstract

Gastric perforation refers to the complete rupture of the gastric wall, leading to the extravasation of gastric contents into the thoracic cavity or peritoneum. Without timely intervention, the expulsion of gastric contents may culminate in profound discomfort, exacerbating the inflammatory process and potentially triggering perilous sepsis. In clinical practice, surgical suturing or endoscopic closure procedures are commonly employed. Magnetic-driven microswarms have also been employed for sealing gastrointestinal perforation. However, surgical intervention entails significant risk of bleeding, while endoscopic closure poses risks of inadequate closure and the need for subsequent removal of closure clips. Moreover, the efficacy of microswarms is limited as they merely adhere to the perforated area, and their sealing effect diminishes upon removal of the magnetic field. Herein, we present a Fe&Mg@Lard-Paraffin micromotor (LPM) constructed from a mixture of lard and paraffin coated with magnesium (Mg) microspheres and iron (Fe) nanospheres for sutureless sealing gastric perforations. Under the control of a rotating magnetic field, this micromotor demonstrates precise control over its movement on gastric mucosal folds and accurately targets the gastric perforation area. The phase transition induced by the high-frequency magnetothermal effect causes the micromotor composed of a mixed oil phase of lard and paraffin to change from a solid to a liquid phase. The coated Mg microspheres are subsequently exposed to the acidic gastric acid environment to produce a magnesium protonation reaction, which in turn generates hydrogen (H_2_) bubble recoil. Through a Mg-based micropower traction, part of the oil phase could be pushed into the gastric perforation, and it would then solidify to seal the gastric perforation area. Experimental results show that this can achieve long-term (>2 h) gastric perforation sealing. This innovative approach holds potential for improving outcomes in gastric perforation management.

## 1. Introduction

The acute perforated peptic ulcer arising from chronic peptic ulceration is a common emergency condition; with associated morbidity rates of up to 50% and mortality in up to 30%, it is regarded as one of the most lethal surgical emergencies worldwide [1]. Gastric perforation, characterized as an acute manifestation of peptic ulcer perforation, denotes a complete breach of the gastric wall, leading to the extravasation of gastric contents into the thoracic cavity or peritoneum [2]. In the absence of prompt intervention, the extrication of gastric contents may culminate in profound nociception, exacerbate inflammatory processes and potentially instigate perilous septicemia [3]. Moreover, hemorrhage may ensue in the vicinity of gastric perforations, with gastric acid impeding hemostatic mechanisms, thereby complicating the healing process substantially and consequently extending the therapeutic regimen [4,5]. Presently, for gastric perforations, clinical interventions typically involve surgical repair [6] or endoscopic closure [7,8,9]. However, surgical interventions carry substantial hemorrhagic risks and protracted convalescence periods, whereas endoscopic closure is constrained by perforation size and location, is ineffective for substantial or deep-seated injuries, and harbors risks of hemorrhage and secondary perforations. Additionally, the retrieval of endoscopic clips necessitates supplementary surgical procedures. In order to prevent gastric perforation from progressing to a level that requires surgery and endoscopic closure, early management of gastric perforation is particularly important. However, conservative management, employing antibiotics and gastric rest, is the only recourse for gastric perforations in early stage, relying on natural closure through supportive measures. It lacks effective targeted measures and is likely to worsen further. Especially for patients with peptic ulcer complicated by bleeding, the risk of rebleeding within 1–2 years is 33%, and within 10 years, the risk of rebleeding is as high as 40–50% [10,11]. After deterioration, traumatic surgery will be required, causing great pain to the patient and requiring a long recovery period. There exists a propensity for progression to peritonitis or abscess formation, with delayed intervention potentially culminating in larger perforations or inciting inflammatory cascades [10]. A new intervention modality remains to be developed.

Recent advancements in micro/nanomotors have rapidly progressed [12,13], finding extensive application in the field of biomedicine [14,15,16,17,18,19,20,21,22,23,24,25]. These motors hold significant promise in various applications such as thrombus clearance in blood vessels [26,27] and facilitating minimally invasive surgeries [28,29,30]. In particular, magnetically driven micro/nanomotors [31,32] emerge as outstanding candidates in biomedicine, leveraging their precise controllability, multifunctionality, wireless manipulation, programmable customized motion patterns, and capabilities for locomotion in both aqueous and terrestrial environments. Moreover, magnetic manipulation enables efficient propulsion of micro/nanomotors within confined spaces or narrow channels [33]. Through magnetic manipulation, these contactless micro/nanomotors can smoothly traverse from the oral cavity through the esophagus to reach digestive sites such as the stomach, facilitating drug delivery to ulcerated areas in ex vivo porcine stomachs for therapeutic interventions [34,35]. In the pursuit of repairing gastrointestinal perforations, Yue et al. present a magnetic-driven microswarm designed for mending micron-scale intestinal perforations [36]. Under the influence of an external magnetic field, the magnetic microswarm maneuver is used to cover and treat micron-scale intestinal perforations. Chen et al. report a multilayer magnetic soft robot for targeted adhesion to gastric ulcers, achieved through magnetic manipulation utilizing hydrogen bonding between adhesive films and moist tissues for ulcer repair [37]. However, the former necessitates sustained magnetic field application to maintain its positioning directly above the perforation site; discontinuation of the magnetic field may lead to misalignment and subsequent leakage of gastric contents. The latter entails a complex fabrication process, and repair at gastric ulcer sites using extensive adhesive layers risks coverage of healthy gastric tissue. Additionally, the adhesive action via hydrogen bonding poses a risk of fluid leakage

Herein, we demonstrate a magnetically driven Fe&Mg@Lard-Paraffin micromotor (LPM) with phase-transition and magnesium-protonation-induced bubble recoil capability for sealing gastric perforations. It comprises biocompatible components including lard, paraffin, iron (Fe) nanospheres and magnesium (Mg) microspheres. By blending two oil phases, the phase-transition temperature can be readily adjusted within the range of 28 °C to 62 °C, thereby regulating it to fall within the tolerable temperature range of the human body (approximately 37 to 42 °C). Additionally, the Fe nanospheres confer magnetic responsiveness and magnetic torque to the micromotor, enabling magnetic control over its movements on gastric mucosal folds. After the magnetothermal effect induces the transition of LPM, it is more likely to block the inside of the perforated area, and can be firmly spread on the surface of the perforated area after solidification. Moreover, in solid LPM, Mg microspheres are wrapped inside, and the oil phase plays a role in blocking water. In liquid LPM, Mg will be exposed on the surface and react with the surrounding environment, so the Mg protonation reaction is controllable to a certain extent. The recoil force generated by the resulting hydrogen (H_2_) gas further enhances the seal’s integrity through Mg-based micropower traction. Experimental results show that this can achieve long-term (>2 h) gastric perforation sealing. This provides a new method and possibility for repairing gastric perforation.

## 2. Materials and Methods

### 2.1. Materials

All the chemicals used in this work were of analytical grade and were used as received without further purification. Commercial Mg microspheres (20 μm) were purchased from TangShan WeiHao Magnesium Powder Co. (Tangshan, China) and washed with acetone twice before usage. Commercial Lard (S26610-1kg) and paraffin (H22585-1kg) was purchased from Shanghai yuanye Bio-Technology Co. (Shanghai, China). Commercial nano iron powder (R018299-25g, 100 nm) was purchased from Shanghai Yien Chemical Technology Co. (Shanghai, China). Sodium dodecyl sulfate (SDS) and methylene blue (MB) were purchased from Sigma-Aldrich (St. Louis, MO, USA). Deionized (DI) water was used in all experiments.

### 2.2. Temperature Control of Paraffin and Lard Mixed Oil

The blending of lard oil with a melting point of approximately 28 °C and paraffin wax with a melting point of approximately 62 °C was conducted in specific volumetric ratios of 3:1.5, 3:1.25, 3:1, 3:0.75, 3:0.5, 3:0.25 and 3:0, respectively. Each blend, totaling 2 mL, was dispensed into seven 5 mL flat-bottomed glass bottles, and subsequently placed on a temperature-controlled heating platform set to 65 °C for uniform heat preservation. Following the removal of the heating platform, the process was meticulously documented utilizing a camera. Furthermore, temperature variations throughout the process were meticulously monitored using a thermocouple thermometer.

### 2.3. Synthesis of LPMs

LPMs were synthesized via a modified emulsification method as shown in Figure S1. The procedure entailed the initial step of introducing 3 mL of a 5 wt% aqueous solution of sodium dodecyl sulfate (SDS) into a 5 mL flat-bottomed glass bottle, labeled as precursor solution No. 1, which was subsequently placed on a temperature-controlled heating platform set at 100 °C for thermal preservation. Concurrently, 1 mL of a mixture containing 20 vol% paraffin (with a volume ratio of lard to paraffin of 4:1) was placed in another 5 mL flat-bottomed glass bottle, denoted as precursor solution No. 2. To this vial, 50 mg/mL of nano iron (Fe) powder and 200 mg/mL of micron magnesium (Mg) powder were added and thoroughly mixed via ultrasonication. The resulting mixture was then preserved on the same temperature-controlled heating platform at 100 °C. Following this, 300 μL of the thermally preserved precursor solution No. 2 was ultrasonically dispersed in a controlled ultrasonic processor (KQ2200DE, Kunshan Ultrasonic Instrument Co., Ltd., Kunshan, China) at 80 °C. The dispersed phase, enriched with the lipid-polymer components, floated atop the surfactant-containing solution. Subsequent vortex mixing (0~4000 rpm) for 30 s facilitated phase separation, allowing for the collection of the lower precipitate layer. This precipitate was then stored in a 5 wt% SDS aqueous solution pre-treated with triple rinsing using ultrapure water before utilization.

### 2.4. Characterization

Scanning electron microscopy (SEM) images were obtained using a Hitachi S-4800 field-emission SEM (Hitachi Co., Ltd., Tokyo, Japan). Motion videos were captured under a bright field using a German Leica microscope DM3000 (Wetzlar, Germany). The gastric surface-related videos were captured under a bright field using a Japanese Olympus stereomicroscope SZX16 (Tokyo, Japan). A powder X-ray diffraction (XRD) analysis of the sample was conducted on the D8 Advanced X-ray Diffraction Meter (l1/41.5418 Å, Bruker, Karlsruhe, Germany). High-frequency-induced magnetic field instruments (Spg400K2, Shenzhen, China) are used in magnetothermal processes as shown in Figure S2. A Millipore Milli-Q purification system was utilized to produce the DI water (18.2 MΩ·cm) used throughout the experiments.

### 2.5. Magnetic Propulsion and Fluid Resistance of LPMs

The magnetically driven experiment was conducted within a customized three-axis Helmholtz electromagnetic coil apparatus affixed to an inverted optical microscope (Leica DM3000B, Wetzlar, Germany) as shown in Figure S3. Initially, 100 μL of LPMs at a concentration of 0.1 mg/mL were introduced into a customized U-shaped microfluidic chip with a channel width of 1 mm and positioned within the operational space of the electromagnetic coil. Subsequently, an alternating magnetic field (B = 10 mT) was applied to the electromagnetic coil via a frequency-adjustable signal generator (NI USB-6343, Austin, TX, USA) and a voltage amplifier (Aigtek ATA-309, Xi’an, China) to induce crawling motion in the LPMs. Motion analysis was conducted using Video Spot Tracker V08.01 software, with statistical analysis performed on a sample size exceeding five LPMs. The process of magnetic propulsion against fluid flow followed a similar methodology, except that pressure gradients were induced on both sides of the microfluidic chip using micro-pumps to drive solution flow. The internal flow velocities within the channels were determined using specialized Particle Image Velocimetry (PIV) V3.60 software.

### 2.6. LPMs for Micropore Occlusion

Assessment of pore occlusion efficacy of LPMs was conducted using Transwell inserts with 12 μm micropores. The Transwell membranes of the four experimental groups were pretreated as follows: (1) blank control group (no treatment), (2) 200 μL 10 mg/mL LPMs without any treatment at room temperature (solid-state LPMs) and dried naturally for 30 min, (3) 200 μL 10 mg/mL LPMs treated with magnetothermal heating (Spg400K2, Shenzhen, China) for 90 s and dried naturally for 30 min then cooled to room temperature and (4) 200 μL 10 mg/mL LPMs with both magnetothermal and simulated gastric acid treatment for 90 s and dried naturally for 30 min then cooled to room temperature. After that, each type of Transwell insert was positioned atop a separate 10 mL beaker containing 6 mL of deionized water. Subsequently, 200 μL of 1.17 mg/mL methylene blue (MB) solution was added to the upper compartment of each Transwell insert, and observations were recorded using a camera.

### 2.7. Magnetic Propulsion on Gastric Surfaces and Pore Occlusion for Gastric Perforation Using LPMs

Gastric surface propulsion was conducted within a customized three-axis Helmholtz electromagnetic coil apparatus, affixed to a stereomicroscope (SZX16, OLYMPUS). Initially, a segment of excised rat gastric surface was obtained. Subsequently, 100 μL of LPMs at a concentration of 0.01 mg/mL were applied to the marginal area of the gastric surface and positioned within the operational space of the electromagnetic coil. An alternating magnetic field was then applied to the electromagnetic coil using a signal generator (NI USB-6343, Austin, TX, USA) and a voltage amplifier (Aigtek ATA-309, Xi’an, China) to induce crawling motion in the LPMs. Motion analysis was performed using Video Spot Tracker V08.01 software. For gastric perforation simulation, a sharp needle tip was utilized to mimic a gastric perforation model on the rat gastric surface. Subsequently, employing the aforementioned magnetic control apparatus, LPMs were precisely directed to the region above the simulated gastric perforation. Heating was achieved for 60 s using a magnetic induction heating device immersed in a simulated gastric fluid. Pre- and post-repair gastric tissue sections were clamped between two equally sized 3 mL tubes, with the upper tube containing 1 mL of 1.17 mg/mL methylene blue (MB) solution and the lower tube filled with 2.5 mL of deionized water to assess repair efficacy for 0 h, 0.5 h, 1 h, 1.5 h and 2 h, respectively. The absorbance at 668 nm was recorded using a spectrophotometer after placing the lower tube for 0 h, 0.5 h, 1 h, 1.5 h and 2 h, respectively. All animal experiments were approved by the institutional Animal Care and Use Committee at Wuhan University of Technology (Reg. No. WHUT2022-008) and performed following European Community Guidelines (2010/63/EU).

### 2.8. Numerical Simulation

The commercial simulation software COMSOL Multiphysics V6.1 was used to simulate the magnetothermal effect and gastric perforation process. Some relevant parameters are shown in Table S1. For the magnetothermal effect, the AC/DC module was used. The system could be solved by

(1) $$j\omega \sigma \left(T\right)A+\nabla \times \left(\mu^{-1}\nabla \times A\right)=0,$$

(2) $$\rho C_{p}\frac{\partial T}{\partial t}-\nabla \cdot k\nabla T=Q(T,A)$$

where *ρ* is the density, *k* is the thermal conductivity, *C_p_* is the specific heat capacity and *Q* is the inductive heating. In the gastric perforation simulation, the two-phase flow module was used to solve the relevant parameters.

## 3. Results and Discussion

### 3.1. Conceptual Design of the LPMs for Gastric Perforation Repair

**Figure 1:**
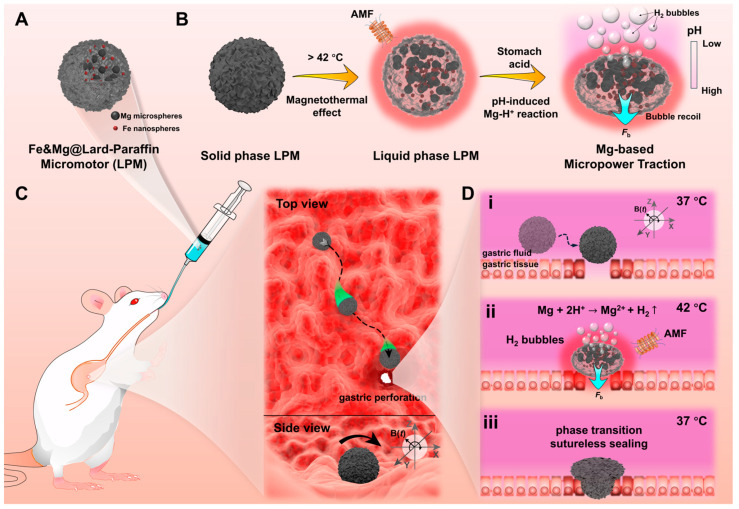
Schematics of a phase-transitional magnetic-driven Fe&Mg@Lard-Paraffin micromotor (LPM) and its application for sealing gastric perforation. (**A**) Schematic of an LPM. LPMs were prepared via emulsion emulsification and formed by coating iron (Fe) nanospheres and magnesium (Mg) microspheres with lard and paraffin mixed oil. (**B**) Schematic process of temperature-induced phase transition and pH-induced bubble recoil. For temperature-induced phase transition: LPM exhibits a solid-state configuration at ambient temperature (20 °C). The magnetothermal effect of Fe nanospheres induces a phase transition in LPM from solid phase to liquid phase under an alternating magnetic field (AMF) when subjected to elevated temperatures (42 °C). For pH-induced bubble recoil, on the acidic side, protons (H^+^) react with Mg to produce hydrogen (H_2_) bubbles that recoil and act on the liquid LPM to deform it through Mg-based micropower traction. (**C**) Upon reaching the gastric cavity of mice, LPM is magnetically propelled with precision across the gastric surface to reach the specified perforation zone. (**D**) Schematic sealing process of the LPM for gastric perforation: (**i**) The LPM is magnetically driven to reach its destination precisely; (**ii**) The magnetothermal effect causes the temperature to rise to 42 °C, causing the LPM to change from solid to liquid phase. At the same time, the coated Mg microspheres can react with protons (H^+^) in gastric acid to produce H_2_ bubbles. The recoil force of the bubbles causes the oil phase to further enter the gastric perforation; (**iii**) After the temperature returns to body temperature (37 °C), LPM changes from liquid to solid phase, and the sealing is completed.

Given the current state, where sealing methods for gastrointestinal perforation via microswarm/micromotor primarily involve surface adhesion [36] or hydrogen bond adhesion [37], there exists a potential risk of gastric content leakage. Herein, we present a magnetically driven Fe&Mg@Lard-Paraffin micromotor (LPM) with phase-transition capabilities for sealing gastrointestinal perforations via mechanical occlusion. In Figure 1, conceptual diagrams pertaining to the magnetic propulsion phase-transition micromotor for gastric perforation sealing are depicted. Initially, LPMs were synthesized via a modified emulsion emulsification method [38], whereby a certain quantity of oil phase containing 100 nm Fe nanospheres and 20 μm Mg microspheres was introduced into a surfactant-containing aqueous solution and homogenized using a vortex mixer. On one hand, the encapsulated Mg acts to neutralize surplus gastric acid around the gastric perforation, while on the other hand, the presence of Mg^2+^ aids in promoting vascular regeneration [39]. The schematic representation of the synthesized LPM structure, as shown in Figure 1A, illustrates the solid-state LPM at room temperature, encapsulating Fe nanospheres and Mg microspheres within it. Figure 1B showcases the intrinsic characteristics of LPMs. Initially, solid-state LPMs undergo temperature-induced phase transition, facilitated by adjusting the oil phase composition to achieve a phase-transition temperature (42 °C) slightly above the physiological temperature of the human stomach (37 °C), owing to the melting point of lard (approximately 28 °C) and paraffin (approximately 62 °C). Additionally, owing to the presence of Fe nanospheres, LPMs exhibit excellent magnetothermal effect, enabling wireless phase transition via an external high-frequency induction device under alternating magnetic field (AMF) (Figure 1B), facilitating controlled phase transition within the stomach environment. Furthermore, upon phase transition in the gastric acid environment, LPMs undergo pH-induced magnesium protonation reactions, generating hydrogen (H_2_) gas, which, through bubble recoil driven by momentum conservation, provides LPMs with a bubble force (*F*_b_) through Mg-based micropower traction (Figure 1B). After elucidating its pertinent characteristics, the specific procedural concept of employing LPMs for phase-transition-based sealing of gastrointestinal perforation is depicted in Figure 1C. Initially, LPMs are orally administered to a mouse model with gastric perforation via passive transport through the oral esophageal route. At 37 °C, LPMs exist in a solid phase, with the outer oil phase serving as a barrier against the external environment, rendering the internal Mg microspheres inert. Subsequently, upon reaching the gastric region, LPMs are wirelessly manipulated on the surface folds of the mouse stomach via an external magnetic field *B*(*t*), enabling LPMs to tumble magnetically and position themselves directly above the gastric perforation site (Figure 1D(i)). Following this, rapid magnetic heating of the Fe nanospheres enveloped within LPMs to 42 °C (a temperature that does not cause damage to normal tissues on a time scale of seconds) is achieved via an external high-frequency induction device. At this point, the LPM undergoes a transition from a solid spherical state to a liquid collapsed state under the influence of gravity, enhancing the probability of internal micron-sized Mg spheres being exposed at the surface of the oil phase. Moreover, in the gastric environment, the acidity is stronger closer to the gastric lumen, leading to a reaction between Mg and the acidic environment, generating bubble recoil propulsion (Figure 1D(ii)), thereby exerting a driving force on the oil phase, compelling it to fill the gastric perforation channel (previous literature has extensively reported on the ability of liquid oil phase-encapsulated Al microspheres to generate propulsion and drive the overall movement of oil phase-based micromotors [40]). Finally, upon cooling from 42 °C to 37 °C, the liquid-state LPMs revert to a solid state, thereby completing the phase-transition-based sutureless sealing and repair of the gastric perforation (Figure 1D(iii)).

### 3.2. Fabrication and Characterizations of the LPMs

**Figure 2:**
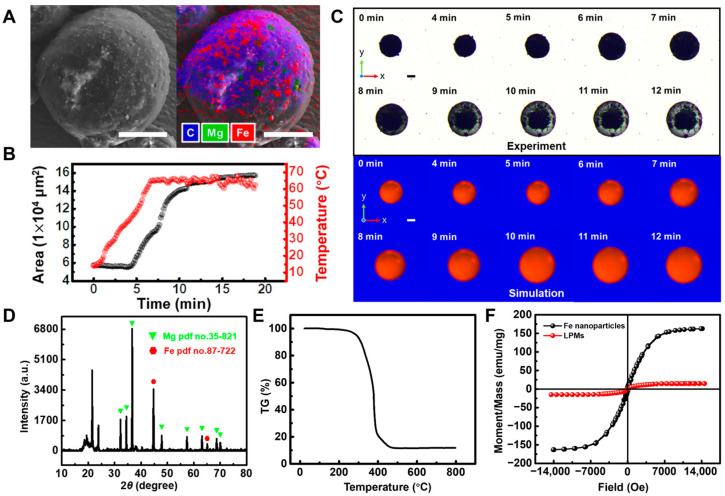
Fabrication and characterizations of the LPMs. (**A**) Scanning electron microscopy (SEM) and energy dispersive spectrometer (EDS) images of a typical LPM, scale bar: 100 μm. (**B**) With increasing ambient temperature, the overlooking area of LPM on a two-dimensional plane changes with time, indicating the occurrence of phase transmission. (**C**) The corresponding time-lapse images of experiment and simulation under a bright field, scale bar: 100 μm. (**D**) X-ray diffraction (XRD) analysis of LPMs. (**E**) The thermogravimetric (TG) curve of LPMs. (**F**) Magnetization curves of Fe nanospheres and LPMs measured form a vibrating sample magnetometer (VSM) at 18 °C.

LPM is an entity with phase transmission capabilities composed of lard and paraffin. The rationale behind opting for lard and paraffin lies in their biocompatible properties. Moreover, commercial lard exhibits a melting point of 28 °C, whereas paraffin melts at 62 °C. By adjusting the temperature of these materials, the melting point of the mixture can be close to 37 °C, facilitating compatibility with physiological conditions. As shown in Figure S4, the mixed oils of paraffin and lard in different proportions were heated to 65 °C simultaneously. At this point, all liquids appeared transparent (as shown in Figure S4A and Video S1). Upon removing the heating platform, the cooling process of the mixed oil with different volume ratios was observed at room temperature, as shown in Figure S4A. It was observed that when the proportion of paraffin reached 20% (i.e., 3:0.75), there was a noticeable tendency for initial solidification at 40 °C. Further analysis through prolonged temperature control as depicted in Figure S4B revealed that at this ratio, the mixed oil was maintained in a liquid phase at 42 °C while presenting a solid phase at 37 °C, aligning precisely with the human body’s gastric temperature, thereby mitigating potential harm. Consequently, employing this ratio of mixed oil, through a modified emulsion emulsification method, led to the synthesis of LPMs. The mixed oil comprises Fe nanospheres of 100 nm (50 mg/mL) and Mg microspheres of 20 μm (200 mg/mL), with their corresponding low- and high-magnification scanning electron microscopy (SEM) images presented in Figure S5A(i–iv), respectively. By controlling the rotation speed of the vortex mixer to 4000 rpm, 3000 rpm and 2000 rpm, LPMs with different diameters of 110 μm, 265 μm and 383 μm were prepared as shown in Figure S5B. At 3000 rpm, the optical microscopy images at low and high magnification are shown in Figure S5C(i,iii), respectively. Further reducing the rotation speed to 200 rpm can prepare macro-millimeter-sized LPM as shown in Figure S5C(ii). Subsequent characterization of the prepared LPMs through SEM and EDS (as shown in Figure 2A) revealed a size of approximately 270 μm, with sparse green spots (Mg element) indicating minimal distribution at the surface of the solid-phase LPM. Subsequently, as illustrated in Figure 2B, external environmental heating resulted in an increase in the LPM area, starting around 42 °C, gradually escalating until reaching a maximum plateau without further increase. This process was further demonstrated under bright-field microscopy in Figure 2C and Video S2, showing good agreement between experimental and simulated results, confirming that temperature increase can induce a phase transition in LPM and increase its covering area by nearly threefold. XRD analysis results (Figure 2D) confirmed the presence of Mg and Fe, while thermogravimetric (TG) analysis (Figure 2E) revealed the presence of mixed oil phases. Further measurement of its hysteresis loop (Figure 2F) demonstrated that LPM containing Fe nanospheres exhibits a good saturation magnetization intensity.

### 3.3. Dynamic Behaviors of LPMs under Rotating Magnetic Field

**Figure 3:**
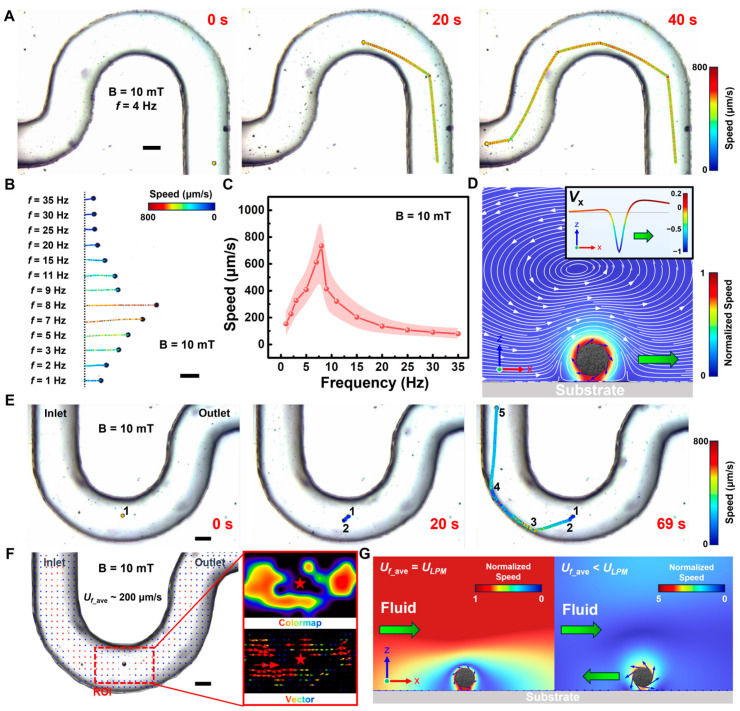
Dynamic behaviors of LPMs under rotating magnetic field (B = 10 mT). (**A**) Under the rotating magnetic field (*f* = 4 Hz), LPM passes through the narrow confined tube in the form of rolling motion. (**B**) The trajectories of LPMs under different magnetic field frequencies during 6.7 s. (**C**) The curve of the relationship between frequency and LPM speed. (**D**) Simulation of magnetic driving mechanism of the LPM. The insert shows the velocity (*V*_x_) distribution on the x-axis on the substrate. The normalized white arrows represent streamline distribution. (**E**) Time-lapse images of LPM for resisting the convection of the fluid over 69 s. (**F**) Particle image velocimetry (PIV) analysis of the average flow velocity (*U_f_ave_*) of the micro-tube. The enlarged region of interest (ROI) images represent the PIV colormap and vector, respectively. The red star represents the relative position of the LPM. The red and blue arrows represent higher and lower flow velocities, respectively. (**G**) Simulations of the flow field distribution around LPM, when *U_f_ave_* is equal to the LPM’s velocity (*U_LPM_*) and *U_f_ave_* is less than the *U_LPM_*, respectively. Scale bar: 1 mm.

Due to the inclusion of Fe nanospheres within LPMs and their favorable magnetic saturation intensity as well as the magnetic moment that a solid LPM can form, they can be propelled under an externally applied magnetic field. Motion modes under magnetic propulsion primarily encompass oscillating, rolling, wobbling, tumbling and spinning [33]. Considering the necessity for motion over uneven gastric surfaces, the rolling mode was a more suitable way to propel. As shown in Figure S6, the LPM can roll accurately under the control of an external magnetic field. In addition to its movement in open spaces, its movement in narrow confined spaces was also studied. As shown in Figure 3, the motion behaviors of LPMs were investigated under a rotating magnetic field. Figure 3A and Video S3 demonstrate LPMs adopting a rolling mode to traverse narrow microfluidic channels under a rotating magnetic field, with LPMs in rolling mode exhibiting an average velocity of approximately 400 μm/s through narrow U-shaped channels. Figure 3B and Video S4 illustrate the trajectories of LPM motion within 6.7 s under different rotating magnetic field frequencies (1–35 Hz). Figure 3C depicts the relationship between magnetic field frequency and LPM motion velocity, revealing that LPMs are out of step after 8 Hz. Furthermore, the driving mechanism was analyzed through simulation, as shown in Figure 3D. The substrate surface flow velocity *V*_x_ in contact with LPMs (inset of Figure 3D) indicates that LPMs, in rolling mode, drive fluid movement near the substrate, thereby propelling their own motion. Additionally, considering the likelihood of gastric content leakage in gastric perforation areas, the influence of microflow was further analyzed on LPM motion, as shown in Figure 3E and Video S5. Phases 1–2 depict LPM resisting fluid to exhibit hovering, phases 2–3 show LPM resisting fluid obliquely, phases 3–4 demonstrate LPM adhering to the wall and phases 4–5 depict LPM resisting fluid in the vertical direction. Further analysis of hovering motion, as shown in Figure 3F through particle image velocimetry (PIV) analysis, reveals an average flow velocity (*U_f_ave_*) within the tube of approximately 200 μm/s, with higher velocities in the central region (indicated in red) and lower velocities at the edges (indicated in blue), consistent with non-slip boundary conditions. Subsequent frame-by-frame PIV analysis of the region of interest (ROI) reveals flow velocities surrounding LPMs close to 0, consistent with corresponding colormap and vector map results. As shown in Figure 3G, we further conducted simulation validation, revealing that when flow velocity approaches LPM velocity (*U_f_ave_* = *U_LPM_*), flow velocities around LPMs decrease to 0, maintaining relative inhibition (phases 1–2). When *U_LPM_* > *U_f_ave_*, the flow field around LPMs exhibits certain velocities, thereby driving LPM motion (corresponding to phases 4–5).

### 3.4. Phase Transition of LPMs for Micropore Repair

**Figure 4:**
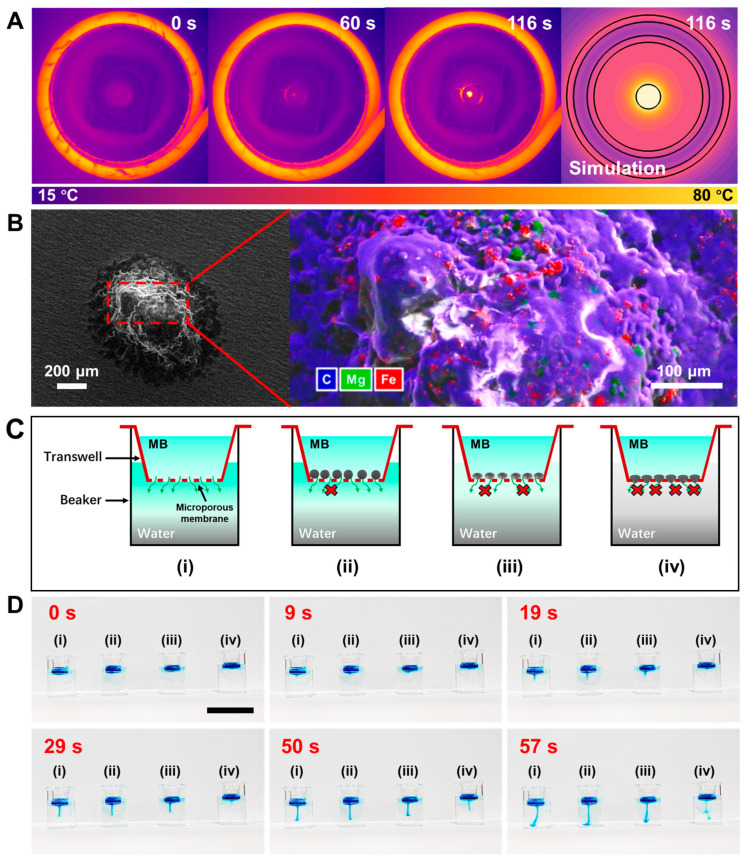
Magnetothermal phase transformation of LPMs with respect to their application for micropore repair. (**A**) Time-lapse images of the magnetothermal effect of LPMs over 116 s. (**B**) SEM and EDS images of a typical LPM after magnetothermal. (**C**) Schematics of the sealing perforation test after pretreatment for repairing micropores: (**i**) control; (**ii**) LPMs without any treatment; (**iii**) LPMs with magnetothermal treatment; (**iv**) LPMs with magnetothermal and acid treatment. The red crosses indicate the degree of obstruction to methylene blue (MB) penetration. (**D**) The corresponding results for repairing micropores and MB are used to show the plugging effect: (**i**) control; (**ii**) LPMs without any treatment; (**iii**) LPMs with magnetothermal treatment; (**iv**) LPMs with magnetothermal and acid treatment. Scale bar: 40 mm.

Upon investigating the magnetic propulsion, we further examined the capability of LPMs to repair micropores. The magnetothermal effect of Fe nanospheres is essential for achieving the phase transition of LPMs, which is first characterized as shown in Figure 4A. A 5 mL centrifuge tube containing 300 μL of 10 mg/mL LPMs was placed in the center of the high-frequency magnetic induction heating coil, revealing that the LPMs containing Fe nanospheres at a concentration of 50 mg/mL could readily attain their phase-transition temperature of 42 °C within 90 s, and further reach temperatures up to 100 °C under continuous magnetothermal actions. In Figure S7, variations in the magnetothermal induction temperature over time under different Fe concentrations are demonstrated. It is observed that in the absence of Fe nanospheres, LPMs do not exhibit magnetothermal effects, maintaining their temperature at room temperature. When the Fe nanosphere concentration is excessively high (100 mg/mL), the temperature of LPMs becomes challenging to control, rapidly rising and surpassing 80 °C between 60 s and 80 s. However, at a concentration of 50 mg/mL, LPMs can be better controlled, with their short-term temperature peaking above 42 °C within 90 s without causing damage to normal tissue cells as shown in Figure S7A. Figure S7B depicts the corresponding time-lapse images of magnetothermal effects within 116 s under Fe nanosphere concentrations of 0 mg/mL and 100 mg/mL, respectively. Considering the optimal temperature range (37 to 42 °C), the heating duration of LPMs via magnetic responsiveness was determined as 90 s, which facilitates phase transition without significant damage to normal tissues. Additionally, in Figure 4B, we characterized the magnetothermally treated LPMs, evidencing that more Mg microspheres encapsulated within were observed on the surface (green dots) of LPMs not inside (only sporadic green spots were observed in its surface EDS in Figure 2A). This indicates that the magnesium microspheres contained inside are gradually exposed to the surface of LPMs after magnetothermal treatment. This results in the solid-state LPM exhibiting inertness in gastric acid conditions (where Mg cannot encounter the external acidic environment), whereas the liquid-state LPM can be driven by bubble recoil generated from magnesium–proton reactions (Figure S8), like the previous reported micromotors driven with oil-phase encapsulated aluminum (Al) microspheres [40].

By utilizing a Transwell model with pore sizes of 12 μm, we simulated the sealing efficacy of LPMs on micropores under various conditions. LPMs with Fe nanospheres at a concentration of 50 mg/mL and Mg microspheres at a concentration of 100 mg/mL were used for the model. We first preprocessed the upper Transwell in Figure 4C, where (i) is the blank control group, (ii) is just added to 200 μL 10 mg/mL LPMs without any treatment at room temperature (solid-state LPMs) and dried naturally for 30 min, (iii) is added to 200 μL 10 mg/mL LPMs treated with magnetothermal heating for 90 s and dried naturally for 30 min then cooled to room temperature and (iv) is added to 200 μL 10 mg/mL LPMs with both magnetothermal and simulated gastric acid treatment for 90 s and dried naturally for 30 min then cooled to room temperature. After that, as shown in Figure 4C, 6 mL of deionized water was kept in the 10 mL beaker below, and 200 μL 1.17 mg/mL methylene blue (MB) aqueous solution was added to the Transwell above at the same time. The corresponding results in Figure 4D and Video S6 indicate that both untreated LPMs and LPMs treated solely with magnetothermal heating exhibit weaker occlusion effects compared to those treated with magnetothermal and gastric acid simulations. The recoil force of bubbles generated by magnesium protonation reactions enables the liquid LPMs to better fill to the pores through mechanical occlusion, thus demonstrating a significant hindrance effect on methylene blue (MB) leakage, most notably at 29 s, where the vertical lengths of leakage in control groups (i), (ii), (iii) are markedly 3.1 to 3.6 times longer than in group (iv).

The infiltration process depicted in Figure 4D may be attributed to two primary factors. Firstly, the excessive quantity of micropores on the surface of the microporous membrane impedes a precise one-to-one correspondence between the LPMs and the pores at a macroscopic scale. Secondly, the morphology of the pores on the microporous membrane, whether they are vertical, slanted or interconnected, also influences the plugging effectiveness of LPMs. The former is an unchangeable fact in the face of hundreds of micropores. The latter we further analyzed using machine learning. Through machine learning combined with bright-field microscopy image analysis, we investigated the distribution status of pores in commercial 12 μm PC membranes. Due to the presence of an angle *θ* between pore penetration and membrane plane, pores are perpendicular when *θ* = 90°. When *θ* ≠ 90°, machine learning methods are required for differentiation. As shown in Figure S9, machine learning enabled us to classify pore morphology, with similar colors representing a single type, delineating approximately eight types due to inconsistent pore angles on the membrane, among which the inclined channels (green) will make it difficult for liquid LPMs to enter them, elucidating the incomplete leakage inhibition observed in Figure 4D(iv).

### 3.5. Actuation of LPMs on Gastric Wall and Repair for Gastric Perforation

**Figure 5:**
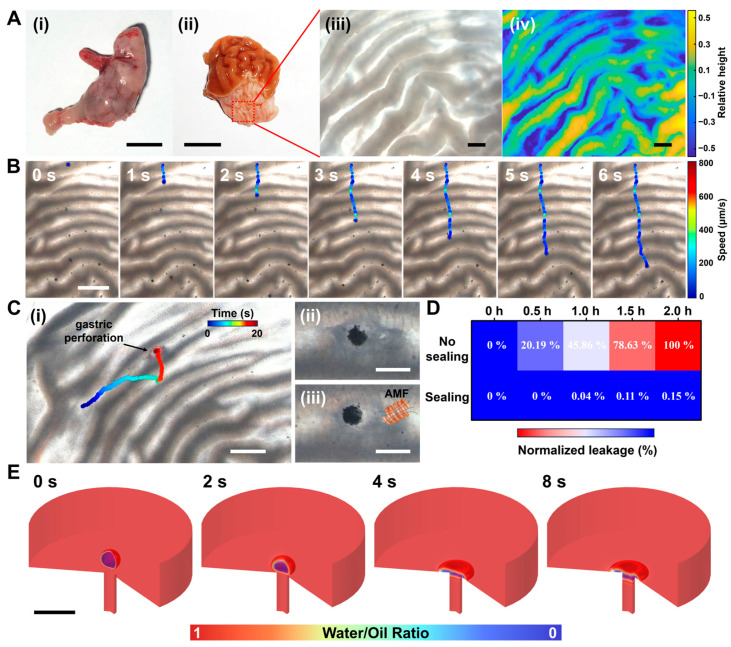
An LPM moves along the gastric wall under a rotating magnetic field and seals a gastric perforation. (**A**) The characteristics of the mouse stomach. (**i**) Image of mouse stomach, scale bar: 1 cm. (**ii**) Anatomical image of the stomach, scale bar: 1 cm. (**iii**) Structural image of the gastric wall under stereomicroscope, scale bar: 1 mm. (**iv**) Gastric fold topography image with the relative height, scale bar: 1 mm. (**B**) Time-lapse images of an LPM moving along on the gastric wall wrinkle over 6 s, scale bar: 2 mm. (**C**) LPM patches up the gastric perforation. (**i**) The LPM accurately reaches the area of gastric perforation in 20 s, scale bar: 2 mm. Before (**ii**) and after (**iii**) magnetothermal and acid treatment, it conclusively sealed the perforation, scale bar: 500 μm. (**D**) Normalized leakage value heatmap via methylene blue (MB) absorption spectrum (668 nm) analysis through microplate reader before and after sealing over 2 h. (**E**) Simulation results of the patching process, scale bar: 500 μm.

We further investigated the role of LPMs in facilitating and repairing gastric perforation processes within the gastric environment. Figure 5A(i) shows a photograph of an isolated mouse stomach. The corresponding anatomy diagram is shown in Figure 5A(ii). The presence of rugged crevices on its surface can be observed. This characteristic becomes more pronounced under stereomicroscopy, as shown in Figure 5A(iii). To obtain a more precise understanding of the relative elevations between its surface peaks and valleys, surface image analysis was employed to delineate their relative heights, as depicted in Figure 5A(iv). Evidently, discernible undulations characterize its surface morphology. Figure 5B and Video S7 illustrate the facile propulsion of the magnetic-driven LPM along these gastric surface folds. Additionally, we analyzed the relationship between the instantaneous velocity variation of LPM during its traversal across gastric surface folds and the corresponding relative heights of the folds. As shown in Figure S10, LPM decelerates while traversing uphill and accelerates while descending. Typically, the instantaneous velocity is at its minimum at the crest and maximum at the trough of the wave. Figure 5C(i) and Video S8 demonstrate the precise positioning of the magnetically controlled LPM, achieving its destination above the gastric perforation within a mere 20 s timeframe. Figure 5C(ii,iii), respectively, illustrate the efficacy of LPM occlusion before and after 90 s of magnetic heating and gastric acid treatment. It is evident that with post-magnetic heating, LPM exhibits noticeable expansion on the surface of the gastric perforation. Subsequently, a comparative analysis of the occlusion effects pre- and post-repair was conducted, focusing on the permeability of the methylene blue (MB) solution. Utilizing a microplate absorbance reader, we assessed the absorbance of the permeated solution in the gastric region with no-sealing and sealing. Remarkably, the experimental findings demonstrate the sustained maintenance of a robust sealed state over a duration extending up to 2 h (Figure 5D). We conducted numerical simulations of this process, as shown in Figure 5E, where the propelling force generated by the reaction of Mg with gastric acid to produce hydrogen bubbles was simplified as a regional volume force (*F*_v_). Employing phase-field methods in simulation, we observed that within 8 s, the LPM could penetrate the interior pores under the combined effects of magnetic heating phase transition and bubble recoil propulsion. Subsequently, transitioning from a liquid to a solid phase, it effectively achieves gastric perforation occlusion, enhancing its repair efficacy. Furthermore, as shown in Figure S11, multiple LPMs are manipulated to seal the same area (within the red dashed box). This provides the possibility of using multiple LPMs to strengthen the same gastric perforation area.

## 4. Conclusions

To sum up, we present a Fe&Mg@Lard-Paraffin micromotor (LPM) composed of biocompatible materials with phase transition and magnetic propulsion capabilities for sealing gastric perforations. Under the influence of an external magnetic field, the solid-state LPM can achieve precise motion along the topographical folds of the gastric wall and reach the designated area above the gastric perforation. Its phase-transition temperature can be controlled within the range of 28 °C to 62 °C by adjusting the ratio of paraffin and lard oils. By regulating its phase-transition temperature to approximately 42 °C, not only does it avoid harming normal cellular tissues in the short term, but it can also rapidly trigger the phase transition from solid to liquid state within 90 s under the magnetothermal effects. The encapsulated Mg microspheres not only neutralize excess gastric acid to promote hemostasis but also promote vascular regeneration through the generation of Mg^2+^. Furthermore, the recoil force generated by the hydrogen gas bubbles enhances the occlusion’s stability through Mg-based micropower traction. With its magnetothermal phase-transition capability and reinforced mechanical occlusion by bubble recoil micropower traction, they can achieve stable sealing for up to 2 h for gastric perforation. This micromotor introduces a “phase-transition” and “Mg-based micropower traction” strategy for biological repair, offering a novel approach for applying the transition of motors from solid to liquid states in the biological domain.

## Data Availability

The datasets generated during and/or analyzed in the current study are available from the corresponding author on reasonable request.

## Supplementary Materials

The following supporting information can be downloaded at: https://www.mdpi.com/article/10.3390/nano14100865/s1, Figure S1: Detailed preparation process of LPMs via modified emulsification method; Figure S2: The corresponding related high-frequency induction device under alternating magnetic field (AMF); Figure S3: Related equipment and software for magnetic manipulation; Figure S4: Mixed oil temperature control by adjusting the proportion of paraffin and lard; Figure S5: Composition characterization of LPMs; Figure S6: Time-lapse images of an LPM steered precisely under a magnetic field over 40 s; Figure S7: Regulation of the magnetothermal effect of LPM with different concentrations of Fe nanospheres; Figure S8: Kinetics of the LPM under simulated gastric fluid conditions at different temperature; Figure S9: Analysis of microplate pore distribution through machine learning (ML). The same color indicates a distribution pattern, scale bar: 200 μm; Figure S10: Gastric fold topography and the corresponding speed distribution comparison chart; Figure S11: Multiple LPMs achieve phase-transitional sealing in the same area of the stomach wall. Table S1: Relevant simulation parameters. Video S1: Natural cooling process of mixed oils with different proportions at 65 °C; Video S2: A typical LPM phase transition process under bright field during heating conditions; Video S3: A LPM passes through the narrow confined tube in the form of rolling motion under the rotating magnetic field; Video S4: The trajectories of LPMs under different magnetic field frequencies during 6.7 s, scale bar: 1 mm; Video S5: A LPM for resisting the convection of the fluid during 69 s; Video S6: The process for sealing micro-holes by LPMs; Video S7: A typical LPM propels forward on the gastric wall wrinkle during 6 s; Video S8: A typical LPM seals the gastric perforation.
