# Supplementary material for: The Model Study of Phase-Transitional Magnetic-Driven Micromotors for Sealing Gastric Perforation via Mg-Based Micropower Traction
Source: Nanomaterials (Basel). 2024 May 16;14(10):865. doi: 10.3390/nano14100865 (PMC11123717; doi:10.3390/nano14100865)
Supplement: Supplementary file 1 [file nanomaterials-14-00865-s001.zip › nanomaterials-2981784-supplementary final.pdf]

nanomaterials

MDPI

Supplementary Materials

The Model Study of Phase-Transitional Magnetic-Driven Micromotors for Sealing Gastric Perforation via Mg-Based Micropower Traction

Kang Xiong and Leilei Xu *

State Key Laboratory of Advanced Technology for Materials Synthesis and Processing, Wuhan University of Technology, Wuhan 430070, China; kangxiong@whut.edu.cn

* Correspondence: xull@whut.edu.cn

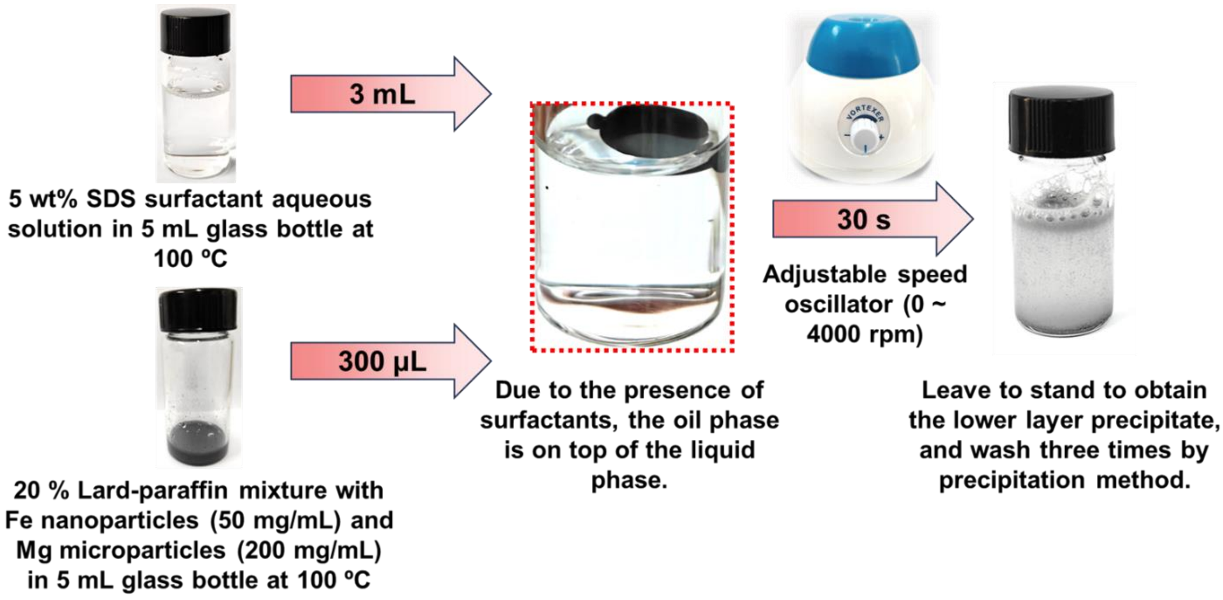

Figure S1. Detailed preparation process of LPMs via modified emulsification method.

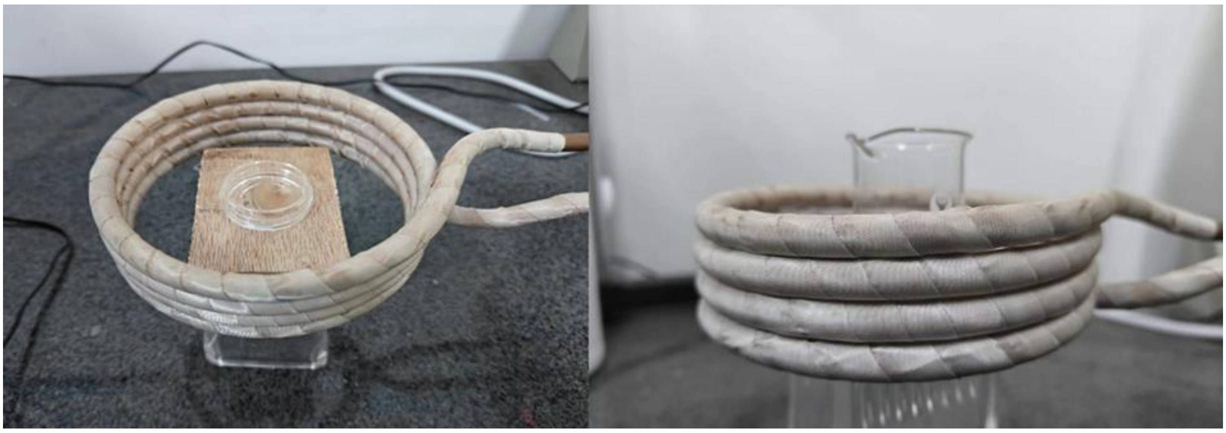

Figure S2. The corresponding related high-frequency induction device under alternating magnetic field (AMF).

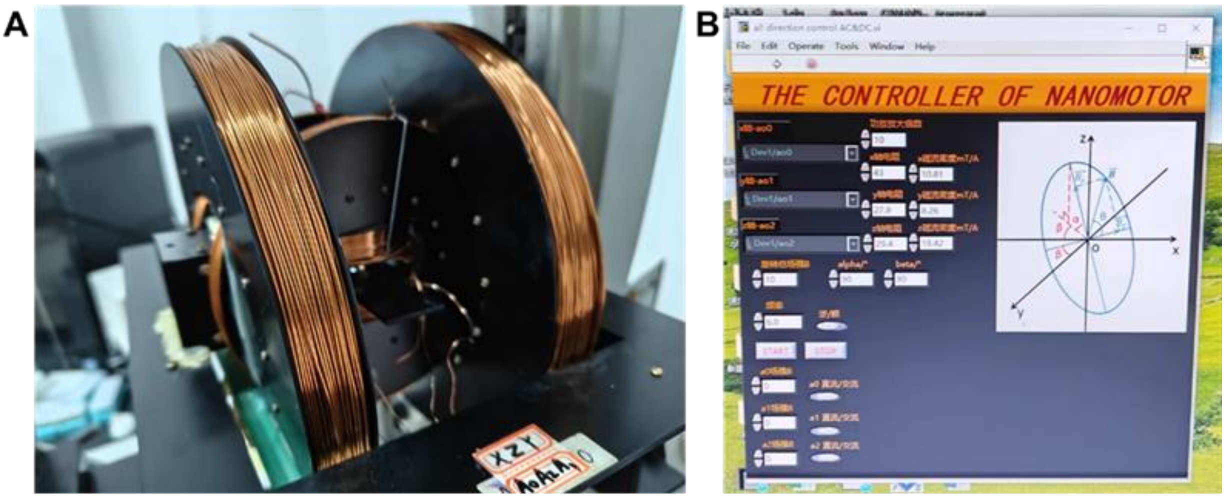

Figure S3. Related equipment and software for magnetic manipulation. (A) A customized three-axis Helmholtz electromagnetic coil. (B) The corresponding control software.

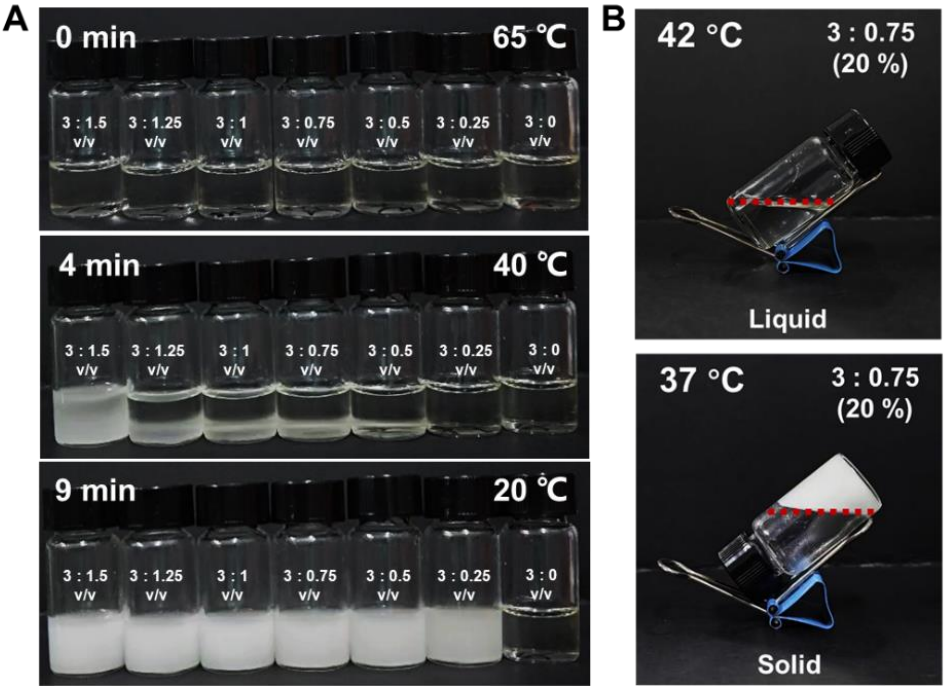

Figure S4. Mixed oil temperature control by adjusting the proportion of paraffin and lard. (A) Natural cooling process of mixed oils with different proportions at 65 °C. (B) When paraffin accounts for 20 vt%, the mixed oil is liquid at 42 °C and solid at 37 °C, respectively.

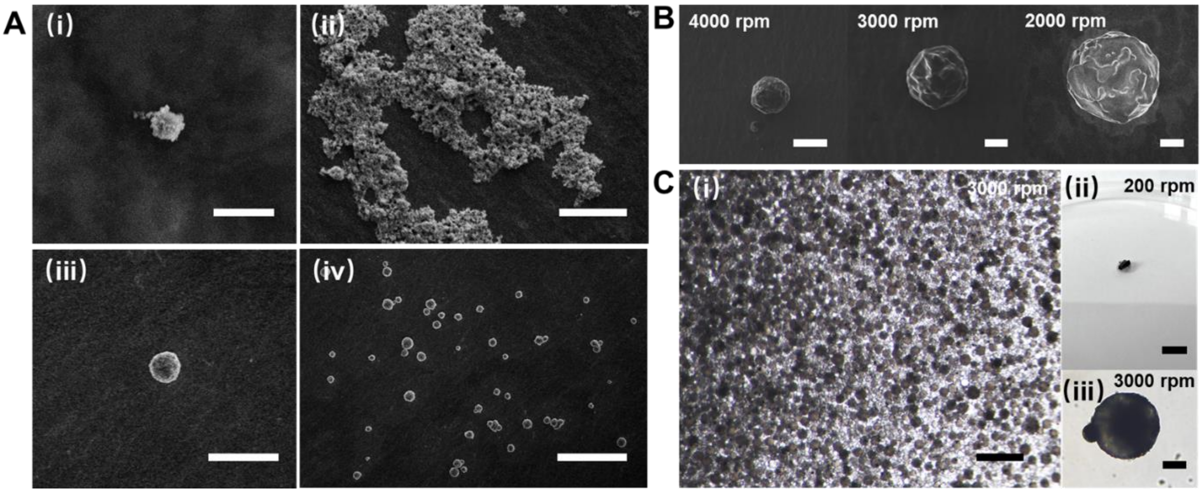

Figure S5. Composition characterization of LPMs. (A) SEM images of Fe nanospheres and Mg microspheres at high (i) (iii) and low (ii) (iv) magnification, scale bar: 500 nm for (i); 50 μm for (ii); 40 μm for (iii) and 100 μm for (iv). (B) SEM images of the LPM prepared at different oscillating speeds (4000 rpm, 3000 rpm and 2000 rpm), scale bar: 100 μm. (C) The microscope image (i) of LPMs prepared at 3000 rpm under bright field, scale bar: 1 mm. (ii) An image of an LPM prepared at 200 rpm, scale bar: 1 cm. (iii) A microscope image of an LPM under a bright field prepared at 3000 rpm, scale bar: 100 μm.

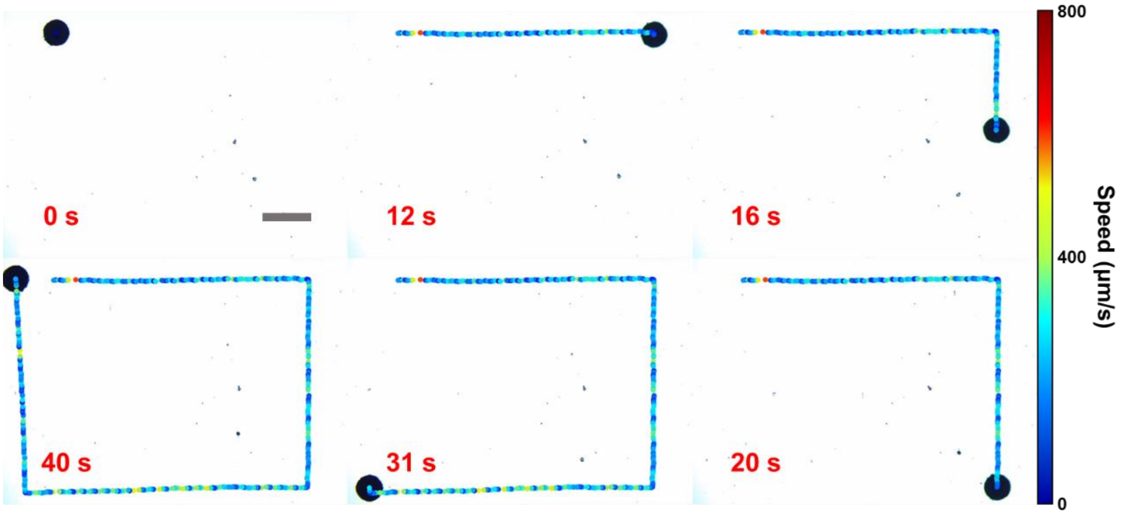

Figure S6. Time-lapse images of an LPM steered precisely under a magnetic field over 40 s.

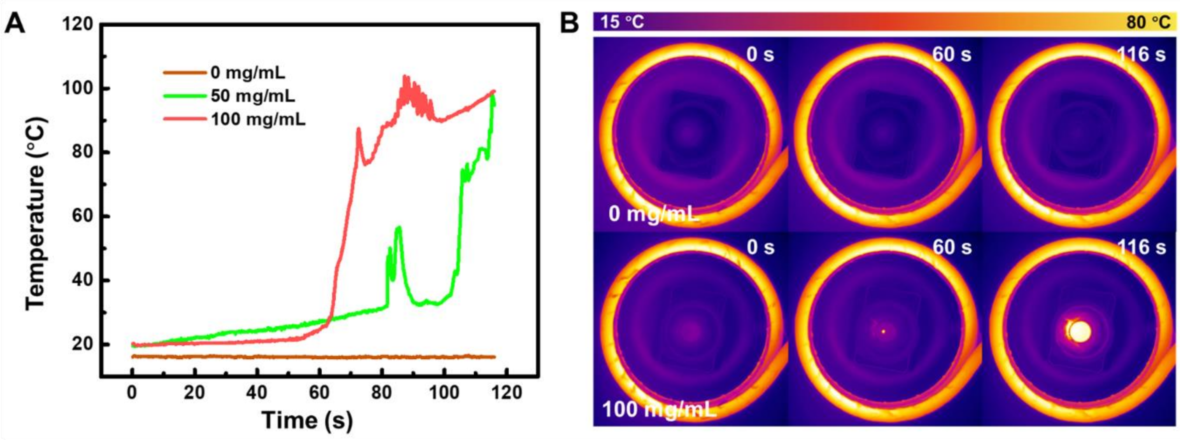

Figure S7. Regulation of the magnetothermal effect of LPM with different concentrations of Fe nanospheres. (A) Magnetothermal temperature changes with time. (B) Time-lapse images of magnetothermal effect of LPMs over 116 s for 0 mg/mL and 100 mg/mL, respectively.

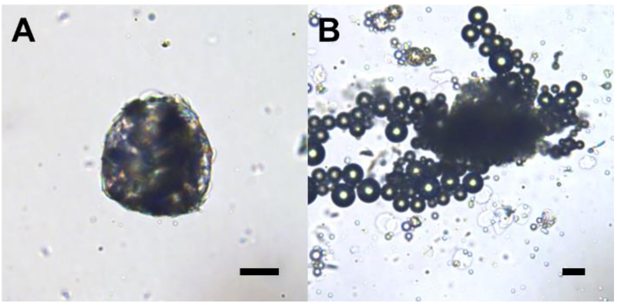

Figure S8. Kinetics of the LPM under simulated gastric fluid conditions at different temperatures. (A) LPM performed at 37 °C under simulated gastric fluid conditions, scale bar: 100 μm. (B) LPM performed at 42 °C under simulated gastric fluid conditions, scale bar: 100 μm.

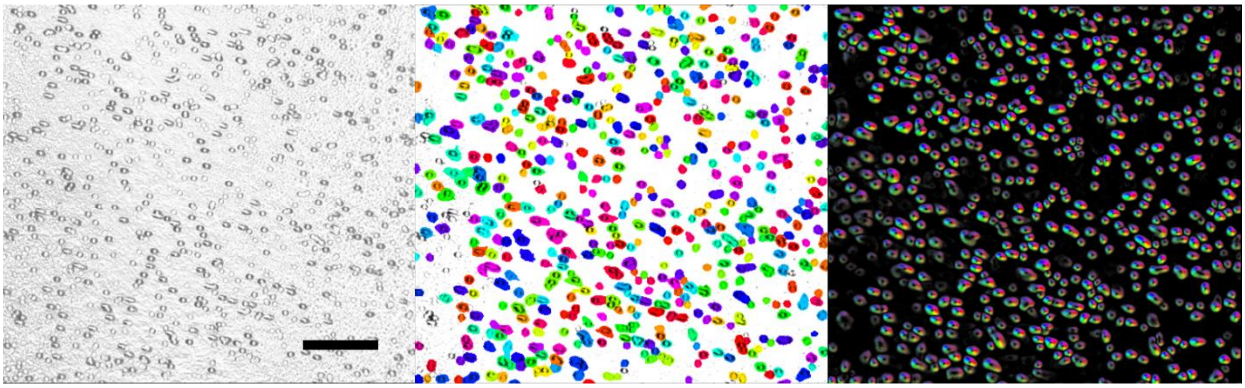

Figure S9. Analysis of microplate pore distribution through machine learning (ML). The same color indicates a distribution pattern, scale bar: 200 μm.

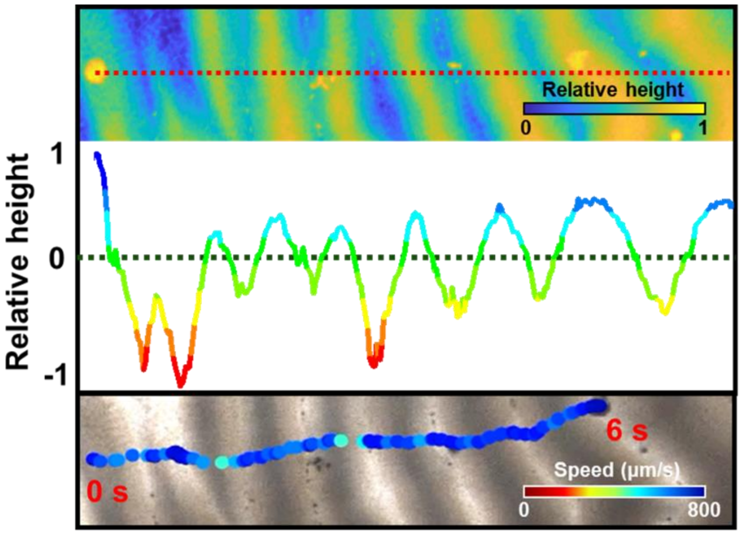

Figure S10. Gastric fold topography and the corresponding speed-distribution comparison chart.

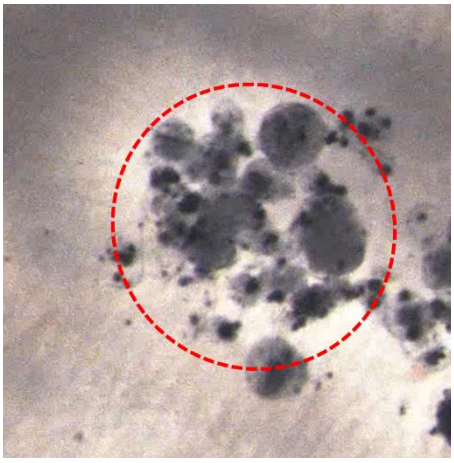

Figure S11. Multiple LPMs achieve phase-transitional sealing in the same area of the stomach wall.

Table S1. Relevant simulation parameters.

Parameters	Expression	Description
I_0	2e3[A]	Current
T_ref	293[K]	Reference temperature
T_in	10[degC]	Cooling water inlet temperature
R_c	5[mm]	Cooling channel radius
A_c	Pi*R_c^2	Cooling channel x-section
M_w	1[kg/min]	Cooling water mass flow rate
r_0	1.75e-8[ohm*m]	Resistivity at T = T_ref
